# High post-treatment serum levels of soluble programmed cell death ligand 1 predict early relapse and poor prognosis in extranodal NK/T cell lymphoma patients

**DOI:** 10.18632/oncotarget.8847

**Published:** 2016-04-20

**Authors:** Hua Wang, Liang Wang, Wen-Jian Liu, Zhong-Jun Xia, Hui-Qiang Huang, Wen-Qi Jiang, Zhi-Ming Li, Yue Lu

**Affiliations:** ^1^ Department of Hematological Oncology, Sun Yat-Sen University Cancer Center, Guangzhou, 510060, China; ^2^ State Key Laboratory of Oncology in South China, Guangzhou, 510060, China; ^3^ Collaborative Innovation Center for Cancer Medicine, Guangzhou, 510060, China; ^4^ Department of Medical Oncology, Sun Yat-Sen University Cancer Center, Guangzhou, 510060, China

**Keywords:** extranodal NK/T cell lymphoma, prognosis, soluble programmed cell death ligand 1, asparaginase, minimal residual disease

## Abstract

The impact of serum levels of soluble programmed cell death ligand 1 (sPD-L1) on prognosis in patients with Epstein-Barr virus-associated malignancies has never been investigated. We prospectively measured pre- and post-treatment serum sPD-L1 levels and evaluated their prognostic value in 97 patients with newly diagnosed, early stage extranodal NK/T-cell lymphoma (ENKTCL) treated with asparaginase-based chemotherapy followed by radiotherapy. For predicting survival outcomes, serum sPD-L1 levels of 3.23 ng/mL and 1.12 ng/mL were respectively identified for pre- and post-treatment cut-off levels. Patients with high pretreatment (>3.23 ng/mL) had shorter progression-free survival (PFS) and overall survival (OS). In a multivariate survival analysis, post-treatment sPD-L1 >1.12 ng/mL, treatment response (complete vs. non-complete response), and stage II disease were independent prognostic factors for shorter PFS and OS. In patients with a complete response, post-treatment sPD-L1 >1.12 ng/mL was associated with shorter PFS and OS. In patients with high pretreatment sPD-L1 levels (>3.23 ng/mL), low post-treatment sPD-L1 level (≤1.12 ng/mL) correlated with longer PFS and OS. Our data suggest the post-treatment sPD-L1 level is a potent biomarker for predicting early relapse and poor prognosis in early stage ENKTCL patients treated with asparaginase, and may be a useful marker of minimal residual disease.

## INTRODUCTION

Extranodal NK/T-cell lymphoma (ENKTCL) is an aggressive non-Hodgkin's lymphoma [1] and is closely associated with Epstein-Barr virus (EBV) infection [2]. ENKTCL is much more common in Asia and Central and South America than in Western countries [3]. Because of the high rate of locoregional and systemic recurrence with standard radiotherapy treatment alone, chemotherapy has been integrated into the management of ENKTCL [4]. There is increasing evidence that asparaginase-based chemo-radiotherapy regimens are superior to anthracycline-based regimens in the treatment of ENKTCL due to overexpression of the multidrug-resistant (MDR) gene in tumor cells [5, 6]. Asparaginase-based chemotherapy combined with radiotherapy, which significantly improves long term survival rates, has therefore become the standard treatment for ENKTCL. Despite recent progresses, nearly 30% of patients with localized ENKTCL still develop local and systemic failures and die due to disease progression [6, 7]. Identification of patients with a high risk of relapse is therefore crucial in ENKTCL treatment.

Immunity response to cancer is also important for controlling tumor progression. However, cancer cells use a number of immune ‘checkpoints’ to evade immune attack [8]. The programmed death 1 (PD-1) receptor protein, which suppresses T cell-mediated immune response, is a key immune checkpoint molecule expressed by T cells [9]. Tumor cells and cells in the local microenvironment both express the PD-1 ligands PD-L1 (B7-H1) and PD-L2 (B7-DC), which bind to PD-1 and inhibit the proliferation of activated lymphocytes and production of cytokines [10, 11]. PD-L1 is upregulated in various types of cancer, and its blockade significantly enhances antitumor effects [12]. Recent studies showed that PD-L1 protein is expressed in tumor cells from ENKTCL patients [13, 14]. Rossille *et al.* recently reported that the soluble PD-L1 (sPD-L1) concentration in blood could predict overall survival and treatment response in diffuse large B cell lymphoma (DLBCL) [15]. In this prospective observational study, we measured sPD-L1 protein levels in pre- and post-treatment serum samples from ENKTCL patients who were treated with asparaginase-based chemotherapy followed by radiotherapy to evaluate the value of this immune checkpoint for predicting survival.

## RESULTS

### Patient characteristics

A total of 97 patients (53 male, 44 female; median age 42 years) were enrolled in this prospective study; patient clinical characteristics are shown in Table 1. Most patients (92 cases, 94.8%) had a favorable performance status (ECOG PS 0–1). Twenty-nine patients (29.9%) displayed B symptoms. Increased LDH levels were observed in 23 cases (23.7%). Forty-one patients (42.3%) presented with regional lymph node involvement. 62.9% (61 cases) of patients in our cohort were positive for EBV-DNA pretreatment. A majority of the patients (87 cases, 89.7%) were classified as low/low–intermediate risk (IPI = 0–1), and 10 patients (10.3%) were classified as intermediate–high/high risk (IPI = 2–5). More patients had KPI = 0–1 (72 cases, 74.2%) than KPI = 2–4 (25 cases, 25.8%).

**Table 1:** Correlation of pretreatment sPD-L1 level with clinicopathological features in ENKTL patients

Characteristics	No	Median sPD-L1 (ng/mL), (range)	*P*-value	Low sPD-L1 group, n (%)	High sPD-L1 group, n (%)	*P*-value
No. of cases	97			63	34	
Age≤60	83	2.93 (0.61–10.45)	0.220	51 (81.0)	32 (94.1)	0.128
Gender (male)	53	2.76 (0.61–10.45)	0.602	33 (52.4)	20 (58.8)	0.670
ECOG PS			0.140			0.050
0–1	92	2.66 (0.61–10.45)		62 (98.4)	30 (88.2)	
≥2	5	4.37 (0.87–4.77)		1 (1.6)	4 (11.8)	
B symptoms (Yes)	29	3.21 (0.72–5.05)	0.122	15 (23.8)	14 (41.2)	0.104
LDH >245 U/L	23	3.27 (0.87-5.87)	0005	10 (15.9)	13 (38.2)	0.023
Stage			0.129			0.164
I	56	2.46 (0.61–10.45)		42 (66.7)	14 (41.2)	
II	41	3.21 (0.72–5.69)		21 (33.3)	20 (58.8)	
Pretreatment EBV-DNA			0.001			0.001
negative	36	1.87 (0.61–2.93)		33(52.4)	3 (8.8)	
positive	61	3.37 (2.09–10.45)		30(47.6)	31 (91.2)	
IPI score			0.04			0.03
0–1	87	2.46 (0.61–10.45)		60 (95.2)	27 (79.4)	
2–5	10	3.56 (0.87–4.77)		3 (4.8)	7 (20.6)	
KPI score			0.021			0.015
0–1	72	2.21 (0.61–10.45)		52 (82.5)	20 (58.8)	
2–4	25	3.27 (0.72–5.05)		11 (17.5)	14 (41.2)	
Treatment response			0.001			0.008
CR	76	2.15 (0.61-5.76)		55 (87.3)	21 (61.8)	
Non-CR	21	3.37 (2.24-10.45)		8 (12.7)	13 (38.2)	
Chemotherapy regimen			0.620			0.639
GELOX	71	2.85 (0.61-5.87)		45 (71.4)	26 (76.5)	
CHOP-L	26	2.43 (0.61-10.45)		18 (28.6)	8 (23.5)	

Abbreviations: ECOG PS: Eastern Cooperative Oncology Group performance status; LDH: lactate dehydrogenase; LN: lymph node; IPI: International Prognostic Index; KPI: Korean Prognostic Index; CT: chemotherapy; CR: complete remission.

### Baseline serum sPD-L1 levels and correlation with clinical features

**Figure 1:**
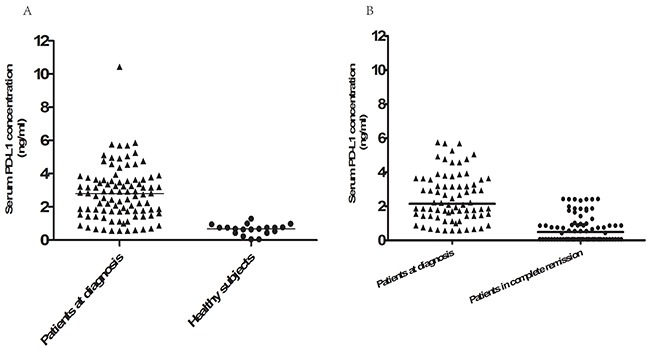
Serum PD-L1 levels in healthy controls and ENKTCL patients both pre- and posttreatment **A.** Serum PD-L1 protein levels in 20 healthy subjects and the 97 ENKTCL patients at diagnosis. Serum PD-L1 levels were higher in ENKTCL patients than in healthy volunteers (*P*<0.001). **B.** Serum PD-L1 protein levels measured at diagnosis and 1 month after the completion of treatment in the 76 patients with complete remission. Pretreatment serum sPD-L1 levels were higher than post-treatment levels in these patients (*P*<0.001).

The median serum sPD-L1 concentration for all patients was 2.76 ng/mL (range, 0.61–10.45 ng/mL), and the mean was 2.79 ng/mL. Serum sPD-L1 was measured in twenty healthy volunteers, and the median of 0.68 ng/mL (range 0.057–1.30 ng/mL) was lower than that of ENKTL patients (*P*<0.001, Figure 1A). ROC curve analysis was used to define an optimal cutoff point for survival prognosis. A cutoff value of 3.23 ng/mL was identified as the most discriminative serum sPD-L1 concentration, with an area under the curve (AUC) value of 0.799 [95% confidence interval (CI) 0.698–0.899, *P*< 0.001]. According to this cutoff value, 63 patients (64.9%) were assigned to the low-sPD-L1 group (≤3.23 ng/L), and 34 patients (35.1%) were assigned to the high-sPD-L1 group (>3.23 ng/L). The baseline clinical characteristics of the patients in the low- and high-sPD-L1 groups are compared in Table 1. The high-sPD-L1 group had a higher proportion of patients with positive pretreatment EBV-DNA levels, elevated serum LDH levels, and high IPI and KPI scores. Serum sPD-L1 levels were higher in EBV-DNA-positive patients and in those with elevated LDH levels, those without complete response (CR) after treatment, etc., as shown in Table 1. However, there was no significant correlation between serum sPD-L1 level and age, ECOG PS, B symptoms, Ann Arbor stage, or chemotherapy regimen. Chemotherapy regimens also did not differ between the low- and high-sPD-L1 groups *(P* = 0.639).

### Treatment response and correlation with post-treatment sPD-L1 level

**Figure 2:**
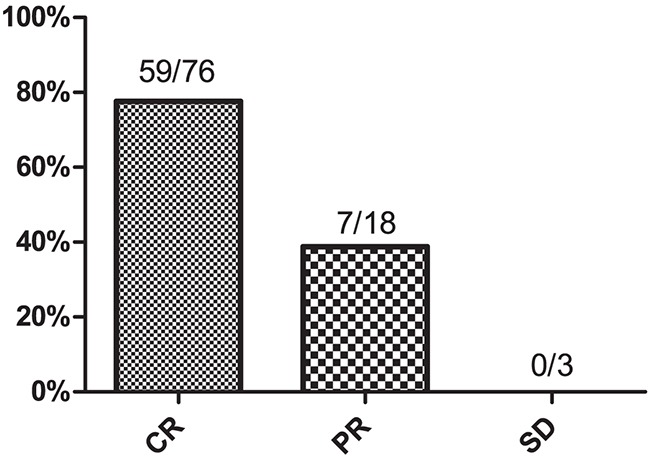
Proportion of patients with post-treatment serum PD-L1 levels≤1.12 ng/mL CR, complete remission; PR, partial response; SD, stable disease.

All patients received asparaginase-based chemotherapy followed by radiotherapy. The GELOX regimen was used in 71 patients (73.2%), and the remaining 26 patients (26.8%) received CHOP-L chemotherapy. After the initial treatment, 76 (78.4%) of the 97 treated patients achieved CR, 18 patients (8.8%) had a partial response (PR), and 3 patients had stable disease (SD). The CR rate at the end of treatment was higher in the low-sPD-L1 group than in the high-sPD-L1 group (87.3 vs. 61.8%, respectively, *P* = 0.008), as shown in Table 1. Post-treatment sPD-L1 levels were lower than pretreatment sPD-L1 levels in the 76 patients who achieved complete remission (*P*<0.001, Figure 1B). We then used ROC curve analysis to determine a cutoff value for post-treatment PD-L1 levels that predicted survival outcome; the cutoff value was 1.12 ng/mL. Post-treatment sPD-L1 levels less than or equal to 1.12 ng/mL were observed in 59 CR patients (77.6%) and 7 patients with PR (38.9%); all SD patients had sPD-L1 levels higher than 1.12 ng/mL (Figure 2).

### Long-term survival and prognostic factors

**Figure 3:**
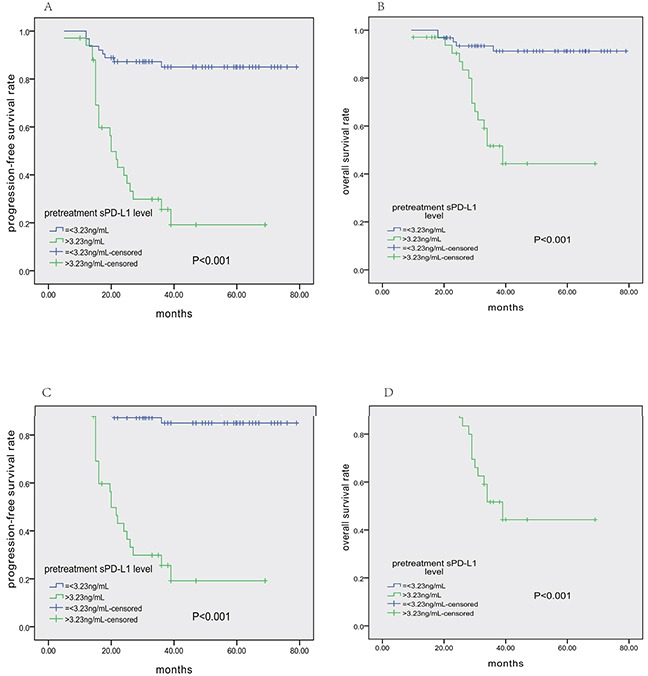
Survival analysis in all 97 ENKTCL patients Pretreatment serum PD-L1 levels>3.23 ng/mL were associated with shorter PFS **A.** and OS **B.** Similarly, lack of CR after treatment also correlated with shorter PFS **C.** and OS **D.**

Within a median follow-up time of 38 months (10–79), the 3-year PFS and OS rates for all 97 patients were 65.0% (95% CI 55.2–74.8%) and 78.8% (95% CI 70.0–87.6%), respectively. Patients in the high-sPD-L1 group had shorter PFS (3-year PFS 85.0 vs. 25.6%, *P*<0.001; Figure 3A) and OS (3-year OS 51.7 vs. 91.3%, *P*<0.001; Figure 3B). Differences in survival were also observed between the patients who achieved CR after treatment and those without CR (both *P*<0.001, Figure 3C, 3D). Survival did not differ between patients receiving GELOX or CHOP-L treatment.

**Figure 4:**
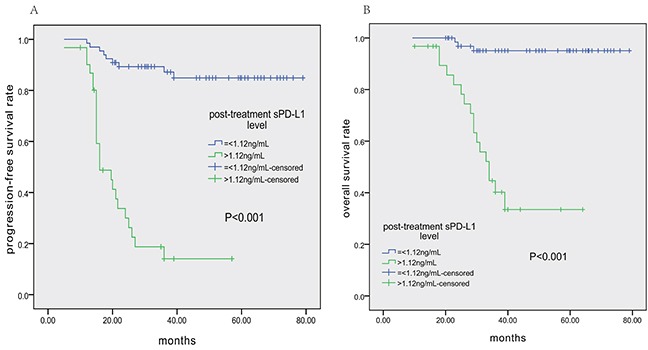
Patient survival outcomes based on post-treatment sPD-L1 levels **A.** Progression-free survival (PFS) according to post-treatment sPD-L1 level (≤1.12 vs. >1.12 ng/mL). **B.** Overall survival (OS) according to post-treatment sPD-L1 level (≤1.12 vs. >1.12 ng/mL).

Table 2 shows the results of univariate and multivariate analyses of prognostic factors for PFS and OS. In the univariate analysis, stage II, pre- and post-treatment EBV-DNA levels, pre-treatment sPD-L1 level>3.23 ng/mL, post-treatment sPD-L1 level>1.12 ng/mL (as shown in Figure 4), and treatment response (non-CR), etc., were associated with shorter PFS and OS. According to the Cox regression model, which did not include serum sPD-L1 levels, post-treatment EBV-DNA level was an independent prognostic factor for both PFS and OS. Interestingly, Cox multivariate analysis including all variables suggested that post-treatment sPD-L1 level>1.12 ng/mL, stage II disease, and treatment response (non-CR) were independent adverse factors for PFS and OS, whereas post-treatment EBV-DNA level lost its prognostic power.

**Table 2:** Univariate and multivariate analysis of factors associated with progression-free and overall survival in all patients

Clinical Characteristics	Progression-Free Survival			Overall Survival		
	Univariate analysis	Multivariate analysis		Univariate analysis	Multivariate analysis	
	*P*-value	RR (95% CI)	*P*-value	*P*-value	RR (95% CI)	*P*-value
Age > 60 years	0.111			0.207		
Gender, male	0.486			0.419		
ECOG PS ≥ 2	0.042			0.044		
B symptoms	0.064			0.115		
Elevated serum LDH	0.001			< 0.001		
Stage II	< 0.001	2.761(1.188-6.416)	0.018	0.003	4.110(1.233-13.695)	0.021
Pre-treatment EBV-DNA level (positive)	< 0.001			0.004		
Post-treatment EBV-DNA level (positive)	< 0.001			< 0.001		
Pre-treatment sPD-L1 level>3.23 ng/mL	< 0.001			< 0.001		
Post-treatment sPD-L1 level>1.12 ng/mL	< 0.001	6.338(2.203-18.232)	0.001	< 0.001	6.515(1.912-22.203)	0.003
IPI score ≥ 2	0.009			0.013		
KPI score ≥ 2	0.002			0.006		
Treatment response (non-CR)	< 0.001	2.918(1.296-6.572)	0.010	< 0.001	7.041(1.540-32.203)	0.012

Abbreviations: PFS, progression-free survival; OS, overall survival; RR, relative risk; CI, confidence interval; ECOG PS, Eastern Cooperative Oncology Group performance status; LDH, lactate dehydrogenase; IPI, International Prognostic Index; KPI, Korean Prognostic Index.

**Figure 5:**
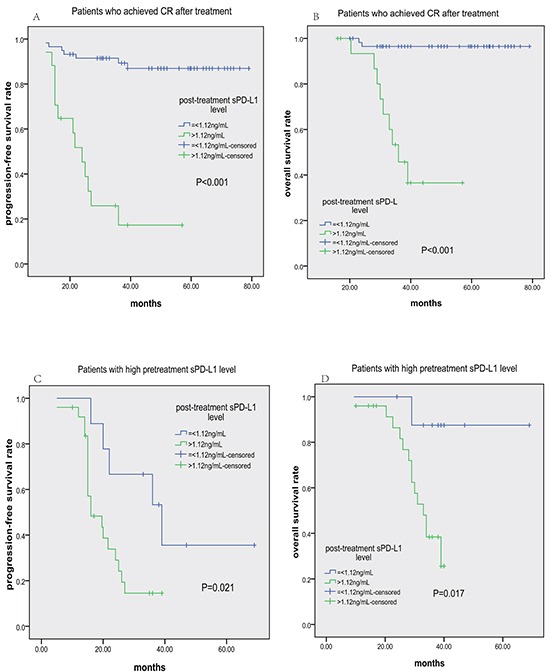
Progression-free survival (PFS) and overall survival (OS) according to post-treatment sPD-L1 level (≤1.12 vs. >1.12 ng/mL) in ENKTCL patients Kaplan-Meier plots of PFS **A.** and OS **B.** for subgroups who achieved CR after treatment; Kaplan-Meier plots of PFS **C.** and OS **D.** for subgroups with high pretreatment sPD-L1 levels (>3.23 ng/mL).

To further assess the predictive value of post-treatment PD-L1 level, we specifically examined its impact on PFS and OS among patients achieving CR after treatment or with high pre-treatment sPD-L1 levels (>3.23 ng/mL). In those achieving CR, patients with post-treatment sPD-L1 levels>1.12 ng/mL had shorter PFS and OS (Figure 5A and 5B, respectively). In the group with high pre-treatment sPD-L1 levels, patients with post-treatment sPD-L1 levels≤1.12 ng/mL had longer PFS and OS (Figure 5C and 5D, respectively). ROC analysis demonstrated that post-treatment sPD-L1 level was more sensitive for predicting tumor relapse than post-treatment EBV-DNA level (72.7% vs. 60.6%, Supplementary Figure S1). Meanwhile, post-treatment sPD-L1 level and post-treatment EBV-DNA level had nearly the same specificity for predicting tumor relapse (89.1% vs. 91.2%). Therefore, post-treatment sPD-L1 level was a better biomarker for predicting tumor relapse than post-treatment EBV-DNA level.

### Immunohistochemical detection of PD-L1 in ENKTCL tumor tissue

We investigated the presence and distribution of PD-L1 in ENKTCL tissue using immunohistochemistry. At least 5% of tumor cells were PD-L1-positive in 76 out of the 97 patients (Supplementary Figure S2). The percentage of PD-L1-positive tumor cells in total malignant cell number was positively correlated with pre-treatment sPD-L1 levels (r=0.8618, *P*<0.0001, Supplementary Figure S3). Patients with high (≥30% of the median value) PD-L1 protein levels had decreased 3-year OS rates compared to those with low (<30% of the median value) PD-L1 levels (60.8% versus 93.4%, *P*<0.001).

## DISCUSSION

In this study of ENKTCL patients receiving an asparaginase-based chemotherapy regimen, we found that high pre-treatment sPD-L1 levels were associated with poor treatment response and post-treatment sPD-L1 level was an independent prognostic factor for both PFS and OS. Specifically, post-treatment sPD-L1 levels identified patients with a CR after treatment who had a high risk of relapse and poor prognosis. Therefore, we propose that post-treatment sPD-L1 levels are a useful biomarker for monitoring minimal residual disease (MRD) in ENKTCL patients.

Previous studies have demonstrated the role of PD-L1 in various solid tumors, including lung cancer, breast cancer, and nasopharyngeal carcinoma [16–19]. A clinical study in adult diffuse large B cell lymphoma patients indicated that higher blood sPD-L1 levels at diagnosis correlated with shorter survival [15]. Epstein–Barr virus (EBV) plays a vital role in the pathogenesis of ENKTL, during which PD-L1 protein levels are high [14]. The EBV-encoded latent membrane protein (LMP)-1 upregulated PD-L1 expression in EBV-associated lymphoma [20]. However, the prognostic role of sPD-L1 in ENKTCL remains unclear. Here, we measured serum sPD-L1 levels in ENKTCL patients and in healthy volunteers. We confirmed that serum sPD-L1 levels were higher in ENKTCL patients and were correlated with clinicopathological characteristics, such ashigher pretreatment EBV-DNA levels, poor PS, elevated LDH levels, and higher IPI and KPI scores. Patients with higher pretreatment sPD-L1 levels may therefore have higher tumor loads and more aggressive tumors.

In this study, elevated serum sPD-L1 levels were associated with poor responses to treatment. In ENKTCL patients, anthracyclines-based chemotherapy, such as CHOP, result in poorer responses because of high MDR gene expression in tumor cells [21]. Our prospective study demonstrated that asparaginase-based chemotherapy (GELOX) improved the CR rate in early stage ENKTCL patients and resulted in longer PFS and OS [6]. However, 20%-30% of ENKTCL patients are resistant to asparaginase-based chemotherapy, and there are no known biomarkers for predicting response to asparaginase-based regimens. Here, patients with high pretreatment EBV-DNA levels had a lower CR rate. We previously measured PD-L1 levels in two ENKTCL cell lines and found they were higher in SNK-6 than in SNT-8 cells. In further *in vitro* studies, we found that ENKTCL cell lines with high PD-L1 expression were less susceptible to pegaspargase and gemcitabine than those with low PD-L1 expression (see Supplementary Figure S4A and S4B). Moreover, cells with high PD-L1 expression had higher levels of Bcl-2 and FasL than cells with low PD-L1 expression (see Supplementary Figure S5). These findings suggest that pretreatment sPD-L1 level may be a useful biomarker for predicting the efficacy of asparaginase-based treatment. Patients with high pretreatment sPD-L1 levels may benefit from novel drugs or more intensive treatments rather than asparaginase-based therapy.

Based on ROC curve analyses, 3.23 ng/mL was an optimal cutoff value for pretreatment sPD-L1 level in determining long-term prognosis in ENKTCL patients. However, our study failed to confirm the independent prognostic value of pretreatment sPD-L1 level on survival outcomes in multivariate analysis when post-treatment sPD-L1 level was added to the final Cox regression model. Many studies have investigated prognostic biomarkers in ENKTCL patients in recent years. IPI, KPI, treatment response, and local tumor invasion were shown to be independent prognostic factors in patients treated with anthracycline-based chemotherapy [22, 23]. However, whether these prognostic factors are still valuable in patients receiving asparaginase-based treatment is unknown. Moreover, these prognostic models are based primarily on pre-treatment clinical characteristics, and a considerable number of patients may experience relapse after achieving CR. Therefore, it is imperative to identify biomarkers that can simply, reliably, and efficiently monitor minimal residual disease. LMP-1 expression in ENKTCL cells upregulates PD-L1 expression on the cell membrane. Soluble forms of ligands are often generated primarily via proteolytic cleavage of membrane-bound proteins, such as sB7-H3 [24]. Here, we found that pre-treatment sPD-L1 levels were positively correlated with the percentage of total malignant cells expressing PD-L1. Post-treatment sPD-L1 level may represent the residual tumor load after asparaginase-based therapy; we found that post-treatment, but not pretreatment, sPD-L1 level was an independent prognostic factor. This indicates that lower residual tumor load, which may reflect the effective eradication of sPD-L1-expressing tumor cell clones during treatment, might be a more important indicator of prognosis than pretreatment tumor load. Moreover, post-treatment sPD-L1 level was a superior biomarker compared to post-treatment EBV-DNA level in that it had a higher sensitivity and similar specificity for predicting relapse. In most patients, a high post-treatment sPD-L1 level may reflect reduced sensitivity to asparaginase-based therapy and insufficient immune response to disease, regardless of whether patients achieved CR, indicating a high risk of early relapse after treatment. Accordingly, patients with high post-treatment sPD-L1 levels had shorter PFS and OS, even if they achieved CR. Similarly, patients with low post-treatment sPD-L1 levels had longer PFS and OS even if their pretreatment levels were high. In conclusion, low post-treatment sPD-L1 levels were associated with more favorable prognoses, regardless of pretreatment sPD-L1 level and depth of response to treatment. We therefore suggest that post-treatment sPD-L1 levels may play an important role in disease monitoring, even for patients who achieve CR after therapy. Future studies should investigate whether alterations in therapy on the basis of these results improve long-term outcomes.

## MATERIALS AND METHODS

### Ethics statement

The ethics committee of Sun Yat-Sen University Cancer Center approved this study; all patients and healthy volunteers signed written informed consent for medical information and serum samples. The study was conducted in agreement with the Helsinki Declaration.

### Eligibility criteria

This was a prospective study in a large cohort of patients newly diagnosed with nasal ENKTCL, who received asparaginase-based chemotherapy followed by radiotherapy at the Sun Yat-sen University Cancer Center between January 2008 and July 2015. The inclusion criteria were as follows: (1) untreated, histologically confirmed ENKTCL patients; (2) primary tumor site located in the upper aerodigestive tract; (3) Ann Arbor stage was IE or IIE; (4) complete treatment and follow-up data; (5) available serum collected at the time of diagnosis and after treatment. The exclusion criteria were: (1) previous or concomitant malignancies; (2) previous anti-cancer treatments; (3) any coexisting medical problems that could cause poor compliance with standard antitumor therapy protocols.

Clinical data collected from patients included the following information: patient demographics, Eastern Cooperative Oncology Group performance status (ECOG PS), physical examination, serum LDH, B symptoms, examination of bone marrow smear, bone marrow trephine biopsy, nasal and oral cavity endoscopic examination, head and neck magnetic resonance imaging (MRI), and positron emission tomography-computed tomography (PET-CT) of the whole body. The standard Ann Arbor staging system was used to determine stage for all patients, as calculated by the International Prognostic Index (IPI: age, PS, LDH level, stage, extranodal sites) and NK/T cell lymphoma Prognostic Index (KPI: LDH level, B symptoms, stage, regional lymph nodes) [23].

### Treatment

All patients were treated with upfront asparaginase-based chemotherapy, e.g. GELOX (gemcitabine, oxaliplatin, L-asparaginase) [7] and CHOP-L (CHOP plus L-asparaginase) [25]. Patients received at least two cycles chemotherapy and a maximum of six cycles. After chemotherapy was complete, a median of 54 Gy radiotherapy was delivered daily at 2 Gy with 5 fractions each week, using three-dimension conformal radiotherapy or intensity-modulated radiotherapy (IMRT). The clinical target volume of limited stage IE patients was defined as the bilateral nasal cavity, bilateral ethmoid sinuses, and ipsilateral maxillary sinus. Above-mentioned zones as well as involved tissues were included in the clinical target volume for extensive stage IE patients. Inclusion of the bilateral cervical lymph node area was required in the clinical target volume for stage IIE disease. Response evaluation was performed after each 2 cycles of chemotherapy or before and after radiotherapy according to standard response criteria for non-Hodgkin's lymphoma [26].

### Serum sample collections and soluble PD-L1 measurement

Blood samples were obtained from the 97 patients at diagnosis and 1 month after the completion of treatment and from 20 healthy volunteers from the Guang Zhou City Blood Bank matched for sex and age with all enrolled patients. Serum was collected from the blood samples by centrifuging at 4000 × g and stored as 500 μL aliquots at −80°C until assays were conducted. Sandwich enzyme-linked immunosorbent assay (ELISA) kits (PDCD1LG1 ELISA kit, USCN Life Science, Wuhan, China) were used to determine serum soluble PD-L1 level according to the manufacturer's protocol. The minimum detectable level of sPD-L1 was 0.057 ng/mL. Briefly, samples and standards were added to a microplate precoated with a PD-L1-specific monoclonal antibody. After enzyme reagent and any unbound antibody was removed by washing, a substrate solution was added to the wells, Stop Solution was used to terminate color development, and the absorbance value was read at 450 nm using a spectrophotometer (Tecan, Mannedorf, Switzerland). The sPD-L1 concentrations were calculated using a standard curve, which was constructed using the standards.

### IHC for PD-L1

Representative formalin-fixed, paraffin-embedded tissues obtained from surgical resections or biopsies were used for IHC. Four-micrometer-thick sections ofparaffin-embedded tissues were cut, placed on slides, deparaffinized in xylene, and hydrated in a graded alcohol series. Immunohistochemical staining of PD-L1 was performed by incubating with a 1:50 dilution of a PD-L1 antibody (ab58810, Abcam, Cambridge, UK). IHC was performed using a modified avidin-biotin peroxidase complex amplification and detection system. PD-L1 expression percentages were quantified by determining the number of cells with membrane staining among the total number of tumor cells in the field examined under high magnification (200x).

### Statistical analysis

Serum sPD-L1 levels are expressed as median (min, max), while categorical data are presented as n (%). Correlations between pretreatment serum sPD-L1 levels and clinical characteristics were analyzed using Mann–Whitney U tests or Chi-square tests. The cutoff levels for pretreatment sPD-L1 and post-treatment sPD-L1 for survival prediction were determined using the receiver operating characteristics (ROC) curve analysis. The Kaplan-Meier method and log-rank test were used for survival analysis. If the two-sided *P* value was <0.05, variables were considered significant in univariate analysis and were entered into multivariate analysis. Multivariate analysis using the Cox regression model was used to evaluate the impact of selected variables on prognosis.

## SUPPLEMENTARY FIGURES


