# Supplementary material for: High post-treatment serum levels of soluble programmed cell death ligand 1 predict early relapse and poor prognosis in extranodal NK/T cell lymphoma patients
Source: Oncotarget. 2016 Apr 20;7(22):33035–45. doi: 10.18632/oncotarget.8847 (PMC5078073; doi:10.18632/oncotarget.8847)
Supplement: Supplementary file 1 [file oncotarget-07-33035-s001.pdf]

High post-treatment serum levels of soluble programmed cell death ligand 1 predict early relapse and poor prognosis in extranodal NK/T cell lymphoma patients

SUPPLEMENTARY FIGURES

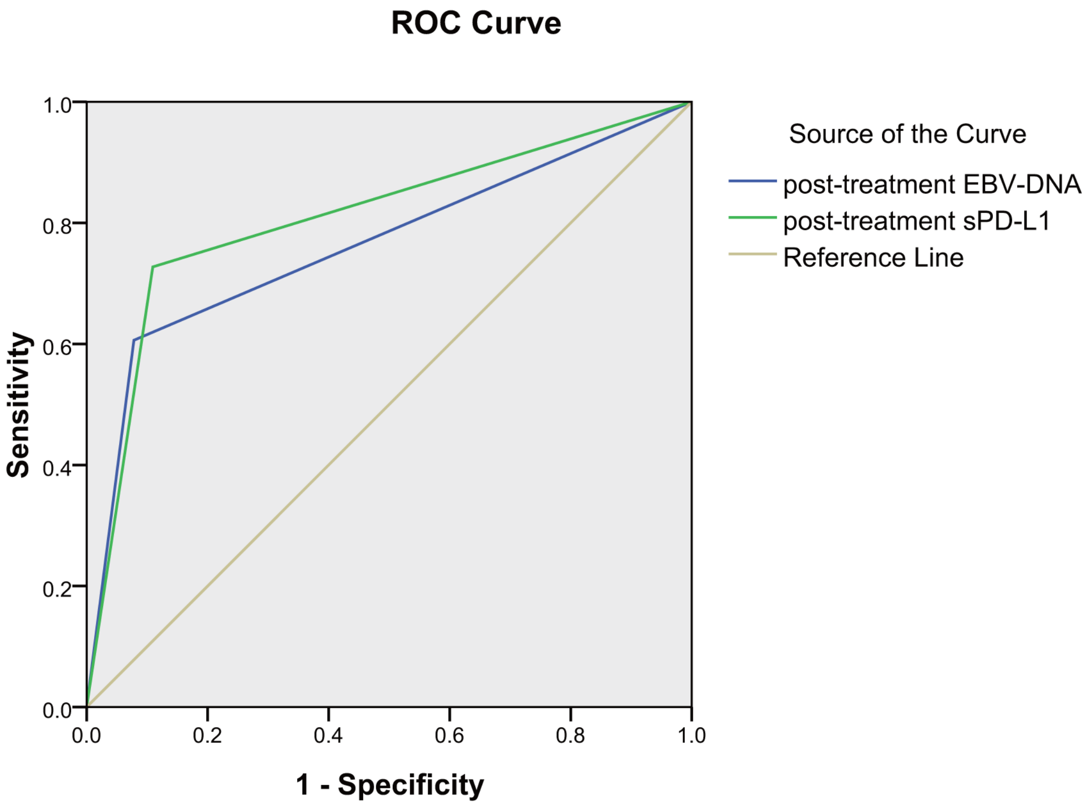

Supplementary Figure S1: ROC curve analysis for the sensitivity and specificity of post-treatment sPD-L1 and EBV-DNA levels in predicting tumor relapse. The sensitivity was 72.7% and the specificity was 89.1% using the cut-off point of 1.12 ng/mL for post-treatment sPD-L1 level, and the area under the ROC curve (AUC) was 0.81 (95% CI, 0.71-0.91). When 0 copy/mL was chosen as the cutoff value, the sensitivity and specificity for post-treatment EBV-DNA level were 60.6% and 91.2%, respectively, with an AUC value of 0.76 (95% CI, 0.65-0.88).

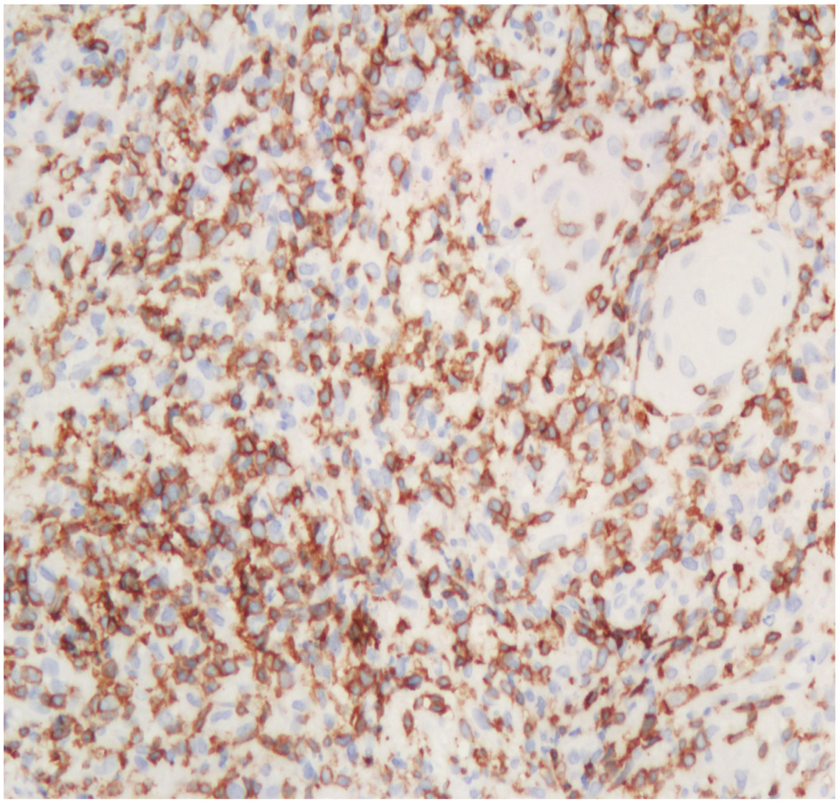

Supplementary Figure S2: Immunohistochemical analysis of PD-L1 expression in ENKTCL patients. A. representative image of PD-L1-positive tumor cells with strong cell membrane staining (brown) is shown (200x magnification).

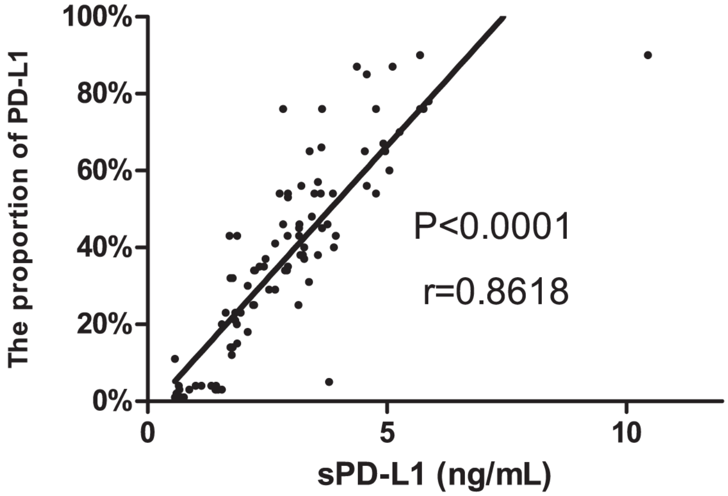

Supplementary Figure S3: Positive correlation between percentage of PD-L1-positive tumor cells and pre-treatment sPD-L1 levels.

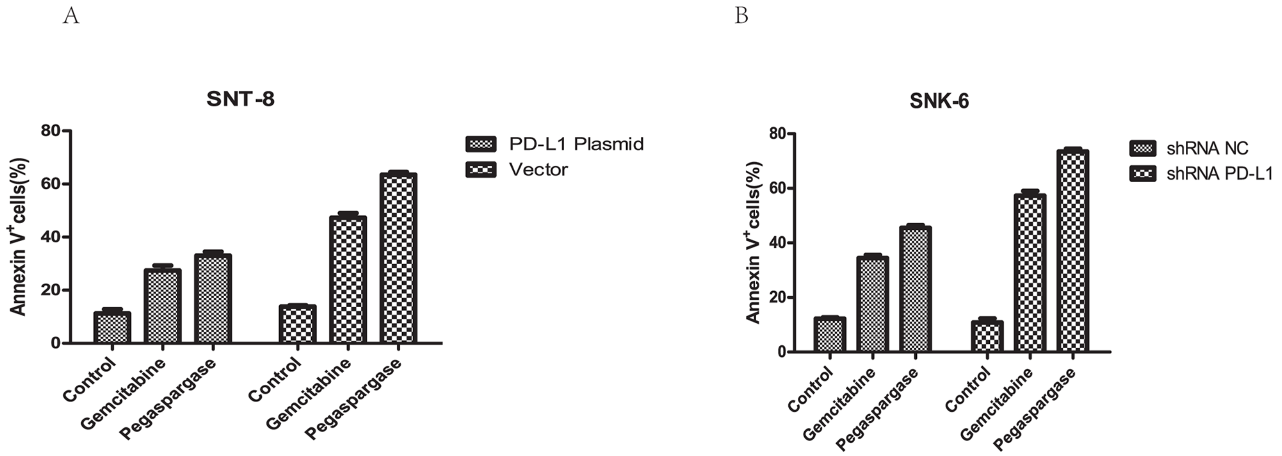

Supplementary Figure S4: Drug sensitivity of high and low PD-L1 expression ENKTL cell lines. A. PD-L1-transduced SNT-8 (PD-L1 plasmid was stably transfected into SNT-8 cell line) and mock-transduced SNT-8 (vector was transfected as negative control) cells were exposed to gemcitabine (40 µg/mL) or pegaspargase (0.01 IU/mL) for 2 days, and annexin-V^+ apoptotic cells in PD-L1-transduced or mock-transduced cells were evaluated using FCM. $P<0.05$, compared with control. **B.** Similarly, shRNA PD-L1-SNK-6 and shRNA NC-SNK-6 (negative control) cells were exposed to gemcitabine or pegaspargase for 2 days, and annexin-V^+ apoptotic cells were detected. $P<0.05$, compared with control.

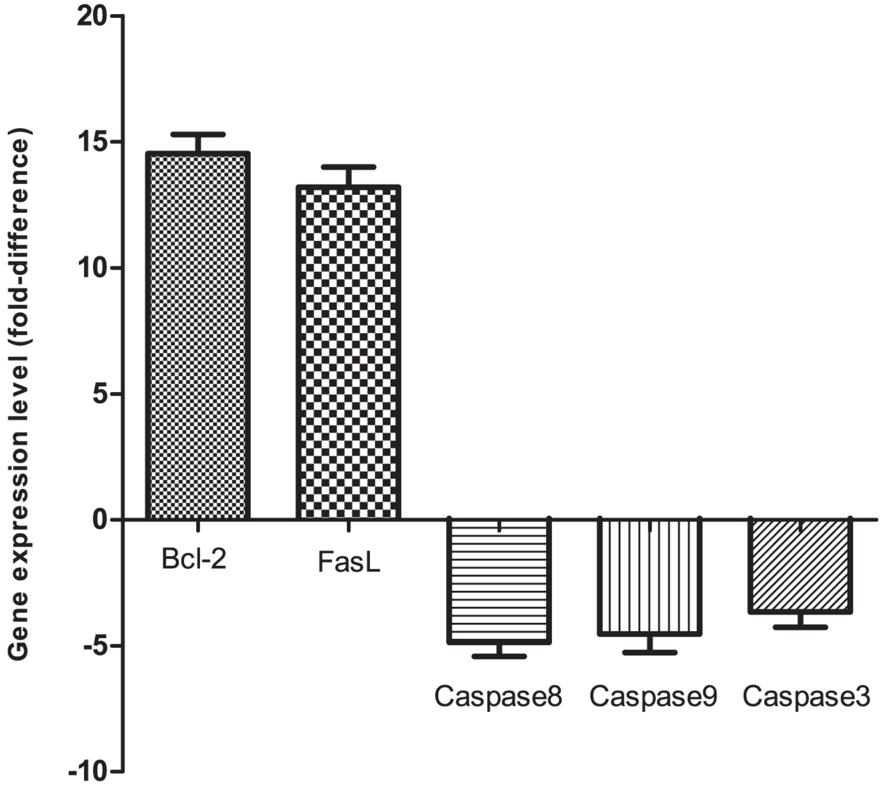

Supplementary Figure S5: Expression of apoptosis-associated genes in PD-L1-transduced SNT-8 cells analyzed using RT-PCR. Data are expressed as fold difference compared to mock-transduced SNT-8 cells.
